# Who Cares for Whom and Is That important? A Typology of Dementia Care Dyads and Health-Related Support Measures

**DOI:** 10.1093/geroni/igaa057.2732

**Published:** 2020-12-16

**Authors:** Karin Wolf-Ostermann, Henrik Wiegelmann, Liane Schirra-Weirich, Lisa-Marie Verhaert, Werner Brannath, Farhad Arzideh

**Affiliations:** 1 University of Bremen, Bremen, Germany; 2 University of Bremen, Bremen, Bremen, Germany; 3 Catholic University of Applied Sciences, Köln, Nordrhein-Westfalen, Germany; 4 Catholic University of Applied Sciences, Koln, Nordrhein-Westfalen, Germany

## Abstract

In Germany, about 70% of all care-dependent persons are community dwelling. In dementia care arrangements you will commonly find a primary informal caregiver (IC) taking on the decisive role in providing care and support for the person with dementia (PwD). This study aimed to develop a typology of dyads based on typical characteristics of the PwD, the IC and their relationship and to gain a better understanding of home-based dementia care arrangements. A latent class analysis was used to detect different dyad types based on personal, social, care and disease characteristics of 551 dyads of CGs and PwD living at home. A 6-class model was identified. The classes could be differentiated based on IC-PwD key characteristics (gender, age, relationship, living situation, occupation). There are significant differences with regard to observed outcomes. The verification of different types of dyads strengthens the need to develop tailored dyad-centred interventions in dementia care

